# Predicting Endoplasmic Reticulum Resident Proteins Using Auto-Cross Covariance Transformation With a U-Shaped Residue Weight-Transfer Function

**DOI:** 10.3389/fgene.2019.01231

**Published:** 2019-12-20

**Authors:** Yang-Yang Miao, Wei Zhao, Guang-Ping Li, Yang Gao, Pu-Feng Du

**Affiliations:** ^1^ College of Intelligence and Computing, Tianjin University, Tianjin, China; ^2^ School of Chemical Engineering, Tianjin University, Tianjin, China; ^3^ School of Medicine, Nankai University, Tianjin, China

**Keywords:** pseudo-amino acid composition, support vector machine, endoplasmic reticulum resident protein, leave-one-out cross-validation, weight transfer

## Abstract

**Background:** The endoplasmic reticulum (ER) is an important organelle in eukaryotic cells. It is involved in many important biological processes, such as cell metabolism, protein synthesis, and post-translational modification. The proteins that reside within the ER are called ER-resident proteins. These proteins are closely related to the biological functions of the ER. The difference between the ER-resident proteins and other non-resident proteins should be carefully studied.

**Methods:** We developed a support vector machine (SVM)-based method. We developed a U-shaped weight-transfer function and used it, along with the positional-specific physiochemical properties (PSPCP), to integrate together sequence order information, signaling peptides information, and evolutionary information.

**Result:** Our method achieved over 86% accuracy in a jackknife test. We also achieved roughly 86% sensitivity and 67% specificity in an independent dataset test. Our method is capable of identifying ER-resident proteins.

## Introduction

The endoplasmic reticulum (ER) is an important subcellular organelle in eukaryotic cells. Two major functions are usually recognized for ER. One is that it selectively transports secreted proteins and membrane proteins. The other is that it retains some proteins to maintain its own structure and function (Lavoie and Paiement, 2008). The ER proteins are sorted precisely with quality controls (Ellgaard and Helenius, 2003; Araki and Nagata, 2011). An understanding of these processes contributes to the elucidation of endoplasmic reticulum function and the pathogenesis of many diseases (Paschen and Frandsen, 2001; Verkhratsky, 2002).

ER-resident proteins are an important topic in ER-related studies. Some of the ER-resident proteins possess sorting signals, such as KDEL or KXXX, while some others do not (Stornaiuolo et al., 2003). Over the last two decades, several efforts have been made to determine the ER sorting signals experimentally. For example, Teasdale and Jackson (1996) found that UGT2 localizes to the endoplasmic reticulum when they studied the UDP-galactosyl transporter (UGT). They also reported that the C-terminal sequence “LLTKVKGS” of the UGT2 is useful in the sorting process. Kabuss et al. (2005) proved that mutating this part of the sequence will result in re-localization of UGT2 to the Golgi apparatus. Although wet experiments for detecting protein localization signals can provide clear evidence and distinguish between maintenance and return signals, performing these experiments is always costly and time-consuming. Therefore, computational predictions are recognized as an alternative approach that provides useful and informative guidance to the experimental methods.

Computational predictions of protein subcellular localizations have been heavily studied in bioinformatics. In the early 1990s, computational systems were developed to recognize the sorting signals from the primary sequences of proteins (Nakai and Kanehisa, 1991; Nakai and Horton, 1999; Wang et al., 2014). When statistical sequence features were introduced to represent protein sequences, machine learning-based algorithms were employed to predict protein sorting destinations. Many studies have tried to apply various algorithms to predict protein subcellular localizations at different levels in different contexts. Several online services have proved useful in this regard. These services include ProLoc-GO (Huang et al., 2007; Huang et al., 2008), KnowPredsite (Lin et al., 2009), SlocX (Ryngajllo et al., 2011), iLoc-Animal (Lin et al., 2013), iLoc-Euk (Chou et al., 2011), Cello v-2.5 (Yu et al., 2006), HybridGO-Loc (Wan et al., 2014), mGOASVM (Wan et al., 2012), Hum-mPloc (Shen and Chou, 2007; Shen and Chou, 2009; Zhou et al., 2017), Euk-mPloc (Chou and Shen, 2007; Chou and Shen, 2010), HPSLPred (Wan et al., 2017), and many others (Chou and Shen, 2008; Briesemeister et al., 2010; Du et al., 2011; Du and Xu, 2013; Almagro Armenteros et al., 2017; Wei et al., 2018; Chen et al., 2019).

The general-purpose protein subcellular location predictors take ER as only one of many subcellular locations. The dataset used for training and testing these methods does not distinguish between ER-resident proteins and non-ER-resident proteins. Since both of these types of proteins may be annotated with subcellular localization ER, constructing a high-quality dataset that is capable of separating them is important. Kumar et al. (2017) proposed the ERPred method, using a carefully curated dataset to distinguish the ER-resident proteins from the non-ER-resident proteins. By using split amino acid compositions (SAAC), they achieved a very promising result. Their results confirmed that the peptide sequences at the terminals of proteins are very informative in guiding the protein sorting process in the ER. Moreover, their results revealed that even if no known sorting signals were found on the sequence, the terminal peptides were still very useful in identifying ER-resident proteins (Kumar et al., 2017).

Pseudo-amino acid composition, which was proposed by Chou (2001), has been widely applied in representing protein sequences for predicting various attributes of proteins. By coupling this with many different machine-learning algorithms, a series of consecutive successes have been achieved. These successful efforts provide consolidated evidence that the pseudo-amino acid compositions are capable of representing protein sequences of various lengths using a fixed-length numerical vector without losing much of the sequential information (Chou, 2011; Chou, 2013; Chou, 2015).

In this study, we introduced a U-shaped weight-adjustment function to improve the pseudo-amino acid compositions. The U-shaped weight-adjustment function transfers weights from the middle-positioned residues to those at the terminals. Besides the weight-adjustment function, we have made two more augmentations to the original pseudo-amino acid compositions. One is to introduce the auto-cross covariance pseudo-factor form, which has been applied in finding protein folding patterns (Dong et al., 2009). The other is to incorporate positional-specific physicochemical properties, which have been applied in predicting protein submitochondrial locations and sub-Golgi locations (Du and Yu, 2013; Jiao and Du, 2017; Zhao et al., 2019).

Our method actually emphasizes the terminal signaling peptide information in pseudo-amino acid compositions. We expect that our approach can be applied not only in predicting ER-resident proteins but also in other topics associated with analyzing protein sorting and localization processes.

## Materials and Methods

### Benchmarking Datasets

In this study, we took the ERPred dataset as our benchmarking dataset. Kumar et al. (2017) released this dataset along with their ERPred study. The ERPred dataset contains two parts: the training set and the independent testing set. **Table 1** gives a breakdown of the entire ERPred dataset. The training set contains 124 ER-resident proteins and 1200 non-ER-resident proteins. The independent testing set contains 65 ER-resident proteins and 2900 non-ER-resident proteins. It is obvious that this dataset is highly imbalanced. The number of non-ER-resident proteins is about 10 times that of the ER-resident proteins in the training set and over 40 times that in the independent testing set. The identifiers of the proteins in the benchmarking dataset are listed in the supplementary materials(**Tables S1**–**S3**).

**Table 1:** Breakdown of the dataset.

Data set	ERRP^a^	non-ERRP^b^
Training set	124	1200
Independent testing set	65	2900

a ERRP, Endoplasmic reticulum resident proteins.

b non-ERRP, Non-endoplasmic reticulum resident proteins.

### Sequence Representations

The ERPred study applied SAAC sequence representations. The result of ERPred implied that the terminal peptides contain more information for sorting proteins to ER (Kumar et al., 2017). Therefore, we introduced a U-shaped weight-adjustment function to transfer weights from those residues in the middle part of the sequence to those at the terminals of the sequence. Besides this improvement, we incorporated the sequential evolution information using the positional-specific physicochemical properties (PSPCP) (Du and Yu, 2013; Jiao and Du, 2017), as well as the auto-cross covariance form pseudo-factors (Dong et al., 2009).

In order to explain our method properly, we developed a new set of matrix-based notations to describe the Type-II classic pseudo-amino acid compositions, also known as the amphiphilic pseudo-amino acid compositions (Chou, 2005). These new formulations, in mathematics, equal the original ones but with a much simpler appearance. We first give the definitions of the all-ones vector and the shifting matrix.

An *n*-D all-ones vector is defined as follows:

(1) $$J_{n}={\left[\begin{array}{cccc} \delta_{1} & \delta_{2} & \cdots & \delta_{n} \end{array}\right]}^{T},$$

where *δ_i_* = 1 (*i* = 1, 2, …, *n*).

An *n*-sized shifting matrix is defined as:

(2) $$M_{n}={\left\{m_{i,j}\right\}}_{n\times n},$$

where

(3) $$m_{i,j}=\{\begin{array}{cc} 1 & ,\text{ when }i-j=1;\\ 0 & ,\text{ otherwise} \end{array}(i=1,2,\mathrm{...},n,j=1,2,\mathrm{..},n)$$

A given protein sequence *p* with length *l* can be represented as a string:

(4) $$p=r_{1}r_{2}\cdots r_{l},$$

where *r_j_* (*j* = 1, 2, …, *l*) is the *j*-th residue on the protein sequence. Every residue represents one of twenty different kinds of amino acids. We use a 20-D binary vector **A** *_j_* to represent *r_j_* (*j* = 1, 2,…, *l*):

(5) $$A_{j}={\left[\begin{array}{cccc} a_{1,j} & a_{2,j} & \cdots & a_{20,j} \end{array}\right]}^{T},$$

where

(6) $$a_{i,j}=\{\begin{array}{cc} 1 & ,\;\text{when}r_{j}\text{ is the}i-th\text{ typeaminoacid};\\ 0 & ,\;\text{otherwise} \end{array}\left(i=\;1,\;2,\ldots ,\;20,j=\;1,\;2,\ldots ,l\right)$$

The whole sequence can be represented using a matrix, as follows:

(7) $$A\left(p\right)={\left[A_{1}\;A_{2}\;\cdots \;A_{l}\right]}^{T},$$

where **A**(*p*) is a matrix-based sequence representation, and **A** *_j_* (*j* = 1, 2, …, *l*) as in Eq. (5).

When the PSSM can be created using the PSI-BLAST program for protein *p*, we can obtain a normalized PSSM scoring matrix for *p*, as elaborated in (Du and Yu, 2013). The normalized PSSM scoring matrix is denoted as follows:

(8) $$B\left(p\right)={\left[\begin{array}{cccc} b_{1,1} & b_{1,2} & \cdots & b_{1,l}\\ b_{2,1} & b_{2,2} & \cdots & b_{2,l}\\ \vdots & \vdots & \ddots & \vdots \\ b_{20,1} & b_{20,2} & \cdots & b_{20,l} \end{array}\right]}^{T},$$

where the following normalization condition is satisfied:

(9) $$\sum_{i=1}^{20}b_{i,j}=1\left(j=1,\;2,\ldots ,l\right)$$

We define the following matrix to combine matrix **B**(*p*) and matrix **A**(*p*):

(10) $$S\left(p\right)=\{\begin{array}{cc} EB\left(p\right) & ,\text{when PSSM can be created for protein}\;p;\\ EA\left(p\right) & ,\text{ otherwise}. \end{array},$$

where matrix **E** is a weight-adjustment matrix. It can be defined as a diagonal matrix, as follows:

(11) $$E=diag\left(\begin{array}{cccc} \varepsilon_{1} & \varepsilon_{2} & \cdots & \varepsilon_{l} \end{array}\right),$$

where *ε_j_* (*j* = 1, 2,…, *l*) is a weight-adjustment factor for the *j*-th residue on the sequence. It is computed by a U-shaped function, as follows:

(12) $$\varepsilon_{j}=l\frac{\exp\left[k\left(2j-l\right)/l\right]+\exp\left[k\left(l-2j\right)/l\right]}{\sum_{j=1}^{l}\left(\exp\left[k\left(2j-l\right)/l\right]+\exp\left[k\left(l-2j\right)/l\right]\right)}\left(j=\;1,\;2,\ldots ,l\right),$$

where *k* is a weight distribution parameter, exp(.) is the exponential function, *l* is the length of the sequence, and *j* is the *j*-th residue.

Given a type of physicochemical property *H*, the values for 20 different types of amino acids can be represented using a 20-D vector.

(13) $$H={\left[\begin{array}{cccc} h_{1} & h_{2} & \cdots & h_{20} \end{array}\right]}^{T},$$

where *h_i_* (*i* = 1, 2,…, 20) is the physicochemical property value of the *i*-th type amino acid. We use the following method to standardize the physicochemical property vector:

(14) $$Ĥ=\left(H-m\left(H\right)J_{20}\right)/sd\left(H\right),$$

where **J**_20_ is a 20-D all-ones vector,

(15) $$m\left(H\right)=H^{T}J_{20}/20,$$

and

(16) $$sd\left(H\right)=\sqrt{H^{T}H/20-m^{2}\left(H\right)}.$$

In this study, we took two different kinds of physicochemical properties into consideration: the hydrophobicity and hydrophilicity of amino acids. We denote them as **H**_1_ and **H**_2_, respectively. We define the sequence auto-cross covariance matrix of physicochemical properties as:

(17) $$R_{u,v}\left(p\right)=S\left(p\right){Ĥ}_{u}{Ĥ}_{v}^{T}S^{T}\left(p\right),$$

where *u*, *v* ∈{1,2}.

The *k*-th order covariance factor can be defined as:

(18) $$\tau_{k,u,v}\left(p\right)=tr\left(R_{u,v}\left(p\right)M_{l}^{k}\right)/\left(l-k\right),$$

where *tr*(.) computes the trace of a matrix, **M** *_l_* the *l*-sized shifting matrix, and *u*, *v* as in Eq. (17). For every given value of *k*, a 4-D covariance vector can be generated as:

(19) $$\theta_{k}\left(p\right)={\left[\begin{array}{cccc} \tau_{k,1,1}\left(p\right) & \tau_{k,1,2}\left(p\right) & \tau_{k,2,1}\left(p\right) & \tau_{k,2,2}\left(p\right) \end{array}\right]}^{T}.$$

By setting the maximum value of *k*, which is denoted as *λ*, we can use a 4*λ*-D vector to contain all covariance factors as:

(20) $$V_{\lambda }\left(p\right)={\left[\begin{array}{cccc} \theta_{1}^{T}\left(p\right) & \theta_{2}^{T}\left(p\right) & \ldots & \theta_{\lambda }^{T}\left(p\right) \end{array}\right]}^{T}.$$

Considering the weight-adjustment factors, the 20-D conventional amino acid composition vector can be constructed as follows:

(21) $$C\left(p\right)=S\left(p\right)J_{l}/l.$$

We can combine the **V** *_λ_*(*p*) and the **C**(*p*) to create a (20 + 4*λ*)-D vector to represent the protein sequence *p*, as follows:

(22) $$F\left(p\right)={\left[\begin{array}{cc} \frac{C^{T}\left(p\right)}{C^{T}\left(p\right)J_{20}+\omega V_{\lambda }^{T}\left(p\right)J_{4\lambda }} & \frac{\omega V_{\lambda }^{T}\left(p\right)}{C^{T}\left(p\right)J_{20}+\omega V_{\lambda }^{T}\left(p\right)J_{4\lambda }} \end{array}\right]}^{T},$$

where *ω* is a balancing parameter between 0 and 1. We use **F**(*p*) to represent protein *p* in this study.

### Prediction Algorithm

We employed a support vector machine (SVM) as the prediction algorithm. The SVM searches for an optimal separating hyper-plane in the high-dimensional feature space, which is widely used in bioinformatics problems (Liao et al., 2018; Meng et al., 2019a; Meng et al., 2019b). The hyper-plane can maximize the margin in the feature space. We applied the radial basis function (RBF) as the kernel function in SVM, because the RBF kernel function is the most flexible and the most widely used of such functions. It can be defined as follows:

(23) $$K\left(F\left(p\right),F\left(q\right)\right)=\exp\left(-\gamma {\left|F\left(p\right)-F\left(q\right)\right|}^{2}\right),$$

where *p* and *q* are two proteins, and |.| is the operator that computes the Euclidean length of a vector.

**Figure 1:**
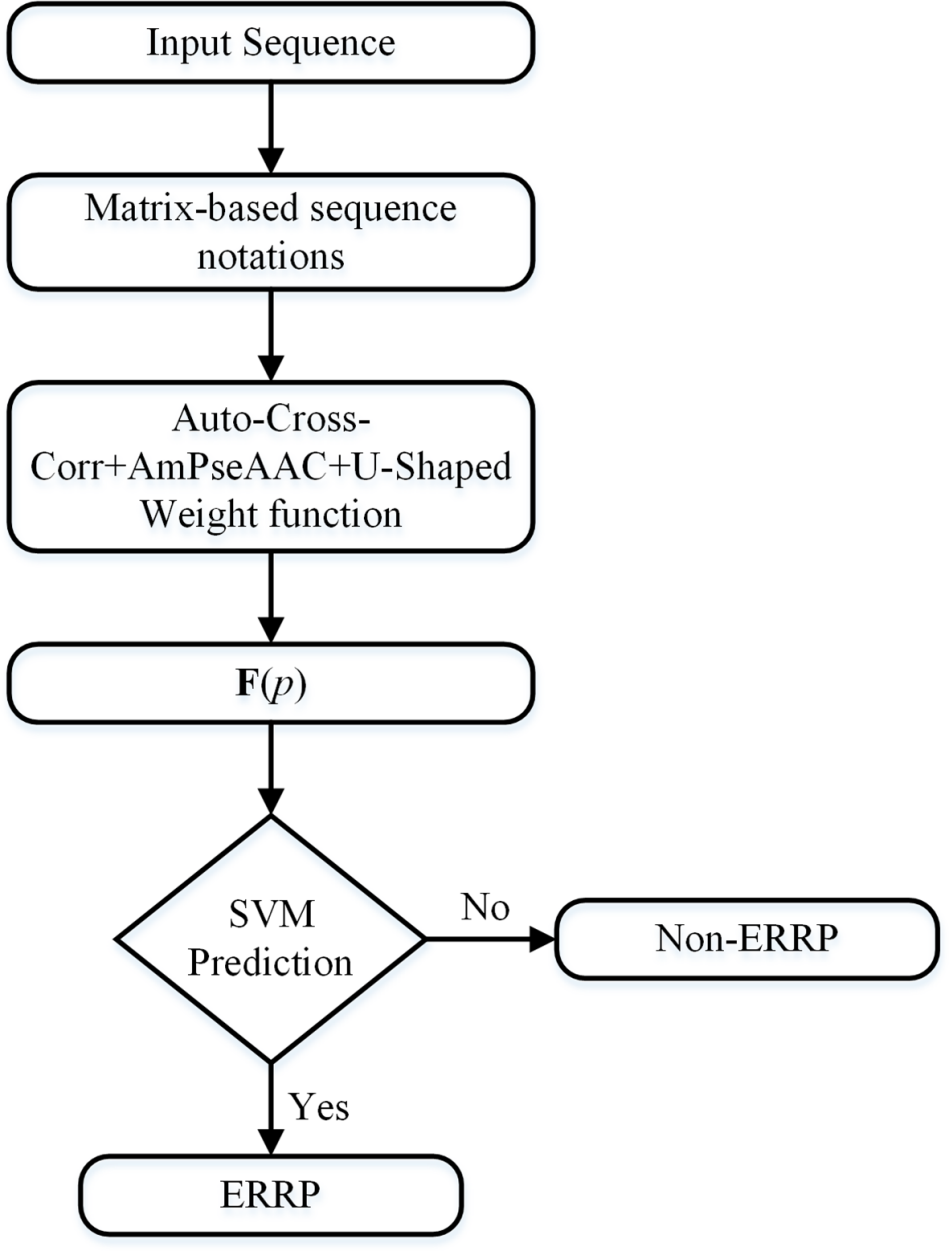
Flowchart of the algorithm. The input sequence will be first converted to matrix-based notations. These notations will be converted into fixed-length numerical vectors, which can represent the sequence order information, the evolutionary information, and the importance of the terminal signaling peptides.

Due to the dataset imbalance, we developed a voting scheme to use all samples in the dataset. We partitioned the negative samples into *m* subsets. The first *m* - 1 subsets have an equal number of negative samples as that of all the positive samples. The remaining subset contains all the remaining negative samples. For each of these *m* subsets, all the positive samples were replicated to compose a training subset. We trained the SVM classifier on each of these training subsets. The final prediction result is the majority result of these *m* classifiers. **Figure 1** is a flowchart of the entire algorithm.

### Evaluation Method

Three validation methods are commonly applied in evaluating a bioinformatics predictor. They are known as the self-consistency test, jackknife test, and independent dataset test (Jiao and Du, 2016). Of them, the jackknife test is usually considered as the most objective and rigorous (Chou and Zhang, 1995). However, some recent studies have shown that the independent dataset test can provide even better estimation to the true performance if a sufficiently large testing dataset can be given (Jiao and Du, 2016). Due to the limited size of the training dataset and the fact that our training dataset is highly imbalanced, we applied the jackknife test to estimate the prediction performance of our method. We also evaluated our method using the independent testing dataset, which allowed us to compare our method to the state-of-the-art methods in a fair manner.

Four statistics were applied to measure the prediction performances of our method quantitatively. They are the sensitivity, specificity, overall accuracy, and the Matthew’s Correlation Coefficient (MCC). They are defined as follows:

(24) $$Sen=\frac{TP}{TP+FN},$$

(25) $$Spe=\frac{TN}{TN+FP},$$

(26) $$Acc=\frac{TP+TN}{TP+TN+FP+FN},$$

(27) $$MCC=\frac{TPTN-FPFN}{\sqrt{\left(TP+FP\right)\left(TP+FN\right)\left(FP+FN\right)\left(TN+FN\right)}},$$

where *Sen* is the sensitivity, *Spe* the specificity, *Acc* the overall accuracy, *MCC* the Matthew’s Correlation Coefficient, and *TP*, *TN*, *FP*, and *FN* are the number of true positives, true negatives, false positives, and false negatives, respectively.

### Parameter Calibrations

Several parameters can be adjusted in our method. The values of these parameters affect the prediction performances. We applied a grid-search strategy to optimize the jackknife test performance by scanning different combinations of the values of *k*, *λ*, and *ω*. The parameter *k* was scanned in the set {0, 0.01, 0.1, 1, 1.5}, the parameters *λ* from 2 to 20 with a step of 1, and the parameter *ω* from 0.05 to 0.95 with a step of 0.05. For each parameter combination, we use another grid-search to find the best values of *c*, *γ*, and *w*, where *c* is the cost parameter of SVM, *γ* is the parameter in the RBF kernel, and *w* is the class weight ratio between two classes. In this study, we applied the SVM functions in the *scikit-learn* python package. The grid search of SVM parameters was conducted automatically with a python script.

## Results and Discussion

### Performance Analysis and Comparison

We obtained the optimized combination of parameters when *k* = 0.1, *λ* = 16, *ω* = 0.55, *c* = 1000, *γ* = 0.01, and *w* = 1.2. The PSSM matrix was created using the PSI-BLAST program with three iterations and 0.001 as the threshold of e-values.

In the jackknife test, our method can correctly identify 111 out of all 124 ER-resident proteins. The prediction performance values are recorded in **Table 2**, with comparison to the ERPred method.

**Table 2:** Prediction performance estimations using a jackknife test.

Methods	Sensitivity	Specificity	Accuracy	MCC
This work	83.1%	86.4%	86.1%	50.6%
ERPred	79.8%	81.6%	81.4%	42.0%

According to these performance values, our method performed better than the ERPred method. Our method achieved a sensitivity of 83.06% and a specificity of 86.38%, which are both higher than the values for ERPred on the same dataset.

### Independent Dataset Test

The training dataset of our work is identical to that used for ERPred. This dataset is highly imbalanced. To further eliminate the concern of over-estimated performances, we performed testing with an independent dataset. We took the same independent testing dataset as used in the ERPred method. The independent testing dataset was processed by the predictor that was trained with the training dataset. The prediction performances of our method are recorded in **Table 3**. Although the specificity is lower than that from the jackknife test, the sensitivity value remains almost unchanged. Therefore, we think the prediction performance is not over-estimated.

**Table 3:** Prediction performance comparison using the independent dataset.

Methods	Sensitivity	Specificity
This work	85.7%	67.2%
ERPred	72.3%	83.7%
Cello 2.5	16.9%	99.9%
iLoc-Euk	15.4%	99.8%
Euk-mPloc 2.0	66.2%	99.0%

We also entered the same testing dataset into several other predictors for comparison. The compared predictors include ERPred (Kumar et al., 2017), Cello v2.5 (Yu et al., 2006), iLoc-Euk (Chou et al., 2011) and Euk-mPLoc 2.0 (Chou and Shen, 2007; Chou and Shen, 2010), which all provide the option to identify ER proteins. According to the prediction performance values, our method has the best sensitivity. However, the specificity of our method is lower. The results indicate that Cello and iLoc-Euk tend to assign non-ER locations to an ER-resident protein. They increase the specificity by severely sacrificing the sensitivity. As the nature of the ER-resident proteins is that the number of non-ER resident proteins is much larger than the resident ones, we think it is acceptable to sacrifice some specificity for the balance to the sensitivity. The ERPred method, Euk-mPLoc 2.0, and our method have a better balance between sensitivity and specificity. Particularly, it seems that the Euk-mPLoc 2.0 method has the best performance, as it achieves over 66% sensitivity while maintaining over 99% specificity. However, it should be noted that Euk-mPLoc 2.0 is not specifically designed to identify ER-resident proteins. Some of the proteins in the testing dataset may have already been used as training samples when Euk-mPLoc 2.0 was developed. This may result in an over-estimated performance value in the comparison. Another factor that should be noticed for Euk-mPLoc 2.0 is that it relies on GO annotations, which makes it not an *ab initio* predictor. Although using GO annotations is common in developing this kind of predictor (Du and Xu, 2013), comparing an *ab initio* predictor with a homology search-based method is not a fair comparison. Therefore, we believe that our method has, at least, comparable prediction performance to other existing methods. Especially in identifying ER-resident proteins, our method should be considered with a higher priority than general-purpose subcellular location predictors.

### Effects of the Residue Weight-Transfer Function

The ER-resident proteins can be roughly divided into two different types. One type is proteins with a specific C-terminal tetra-peptide signal, which usually has a form like KDEL or HDEL. The other type is proteins without this kind of signaling peptide on either its C-terminal or N-terminal. The latter types of proteins usually have an N-glycan modification or similar modifications like cereal prolamin storage proteins (Stornaiuolo et al., 2003). In our training dataset, we searched for the tetra-peptide signals by using ProSite. We found only 41 signaling peptides in all of the 124 ER-resident proteins. In our testing dataset, we performed the same search. We found only 11 singling peptides in all of the 65 non-ER-resident proteins. Therefore, it is not practical to identify ER-resident proteins using only the signaling peptide information. This observation is consistent with the motivation of the ERPred study.

**Figure 2:**
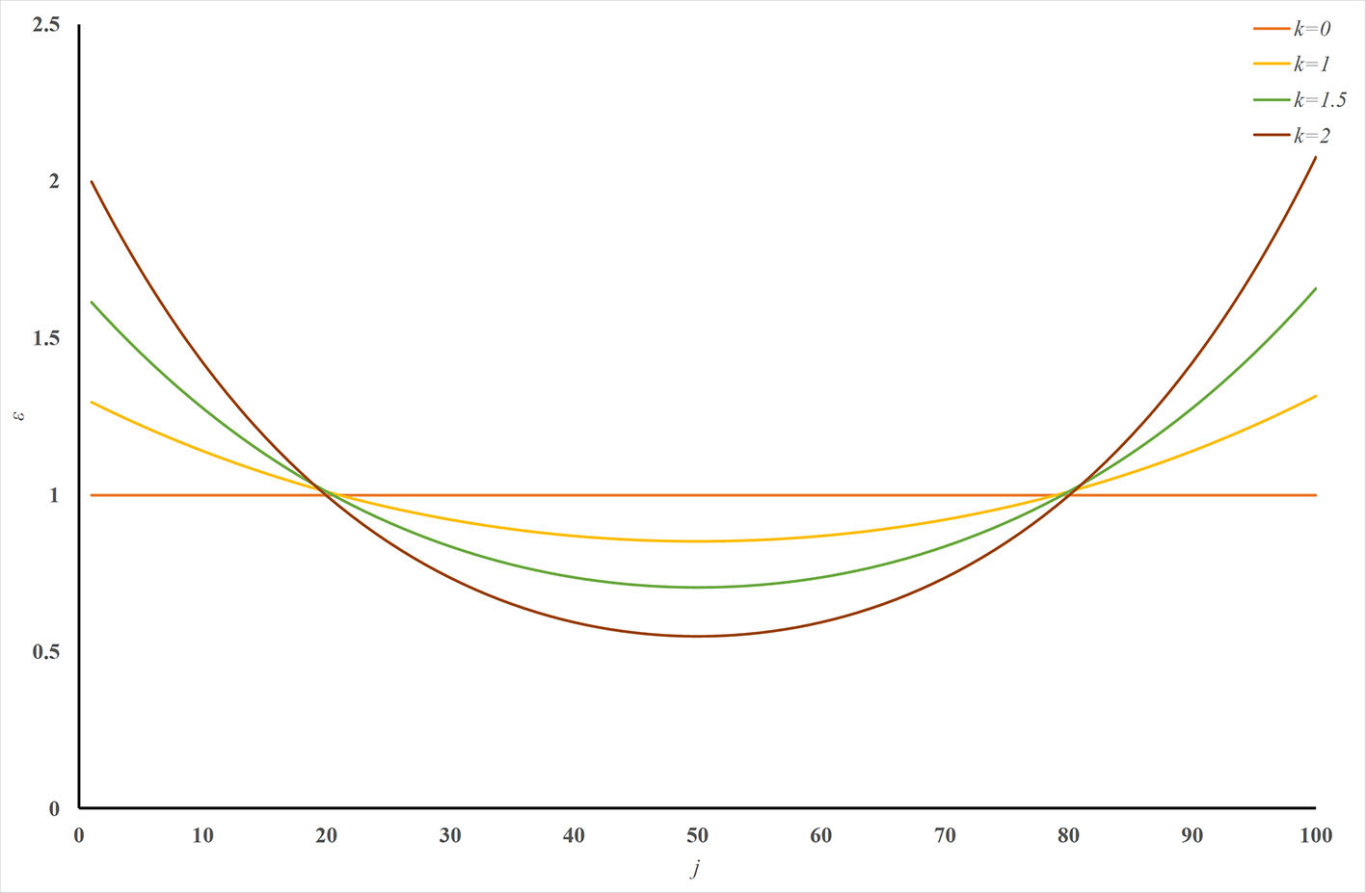
Illustration of the U-shaped weight-transfer function with various *k* values. The U-shaped function transfers weights from the middle part of a sequence to its terminals. The total weight of a sequence does not change after applying the U-shaped weight-transfer function. When the parameter *k* is 0, every residue on the sequence has equal weights, which will produce identical results as where there is no weight-transfer function. When the value of *k* increases, more and more weights are transferred from the residues in the middle part of a sequence to the residues on its terminals.

ERPred is a very powerful and useful computational method. It introduces SAAC sequence representations, which successfully emphasize the terminal signaling sequence information. However, the sequence order information is lost in the amino acid composition representations. Although the pseudo-amino acid composition representation can preserve the sequence order information, it cannot emphasize the terminal signaling peptides in the protein sequence. Therefore, we introduced a U-shaped weight-transfer function into the pseudo-amino acid composition in this study. The purpose of this weight-transfer function is to emphasize the terminal signaling information and also to incorporate the sequence order information. However, it is difficult to decide how many weights should be transferred to the terminals from the middle part of a sequence. We formulate this factor as a parameter *k* in Eq. (12). **Figure 2** illustrates the shape of the function with different *k* values. **Figure 2** enables an intuitive understanding of this U-shaped weighting function. The larger the value of *k*, the more weights are transferred to the terminals of a sequence. Please also note that **Figure 2** is only an intuitive illustration of the U-shaped function when the length of a protein is 100. The crossing point under this condition cannot be extended to other cases.

**Figure 3:**
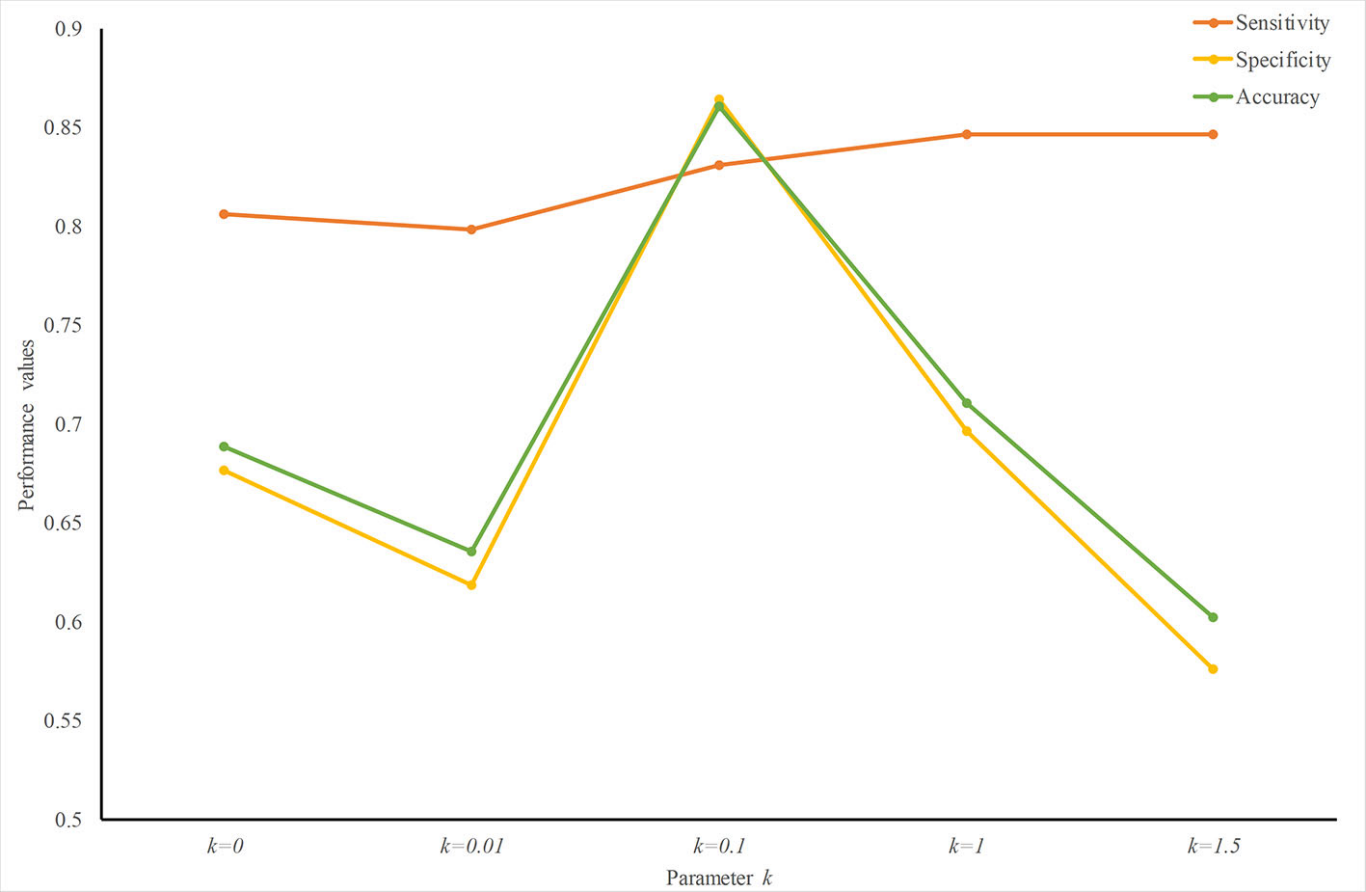
Performance analysis with different weight-transfer functions. Prediction performance varies with the value of parameter *k* in the weight-transfer function. When *k* = 0.1, the performance value peaks. This means that the residues on the terminals are slightly more important than those in the middle part in predicting ER-resident proteins.

To find an optimized *k* value, we trained and tested predictors with different k values. **Figure 3** plots the performance values with different *k*. The sensitivity increases slightly with an increase in *k*. The specificity peaks when *k* = 0.1. Therefore, at least for predicting ER-resident proteins, *k* = 0.1 creates a good weight-transfer function.

The choice of using a U-shaped function rather than another shape is not easy. Since we do not know how much weight should be transferred, this must be an adjustable parameter in the function. Besides, we need to make the function satisfy the following conditions at the same time: (1) all weights are positive; (2) the sum of all weights equals the sequence length; (3) the portion of the weight-increased part and weight-decreased part remains almost unchanged when we adjust the amount of weight that is transferred. This will make the function only transfer weights among residues, not create or remove total weight. The U-shaped function not only satisfies all these conditions but also provides us with a simple way to implement it.

### Sequence Representation Augments

Besides the U-shaped weight-transfer function, we augmented the classic amphiphilic pseudo-amino acid compositions in two ways. One is to use auto-cross correlation to replace the auto-correlation in the classic amphiphilic pseudo-amino acid compositions, while the other is to use matrix-based notations to represent the sequence itself.

The advantage of using auto-cross correlation over auto-correlation has been proved in predicting protein folding patterns (Dong et al., 2009). The matrix-based sequence notations see each residue on the sequence as a 20-D composition vector. The original sequence can then be represented using the one-hot encoding scheme, which can be unified with the normalized PSSM. Since PSI-BLAST cannot generate PSSM for every protein sequence, the matrix-based notation actually provides a mathematically compatible way to compensate for the missing PSSM using the one-hot encodings. As elaborated in Du and Yu (2013), when the PSSM is available for a protein sequence, this matrix-based notation also adjusts the weights of residues according to the evolutionary information.

Therefore, our sequence representation actually encoded the sequence order information and the evolutionary information with emphasis on the terminal signaling peptides in a (20 + 4*λ*)-D numerical vector. Compared to other studies, our sequence representation has a much lower number of dimensions. On a dataset with limited samples, the risk of over-estimated performance increases with the number of dimensions of the representation. Our method should be a better choice when the number of samples is limited.

## Conclusions

Many existing methods can predict protein subcellular locations. However, only the ERPred method can specifically identify ER-resident proteins. The ER may be the most important type of subcellular organelle, linking all the major subcellular structures, including the nucleus, cytoplasm, and cell membrane. In this study, we present a new method for predicting ER-resident proteins. Although establishing a web server for a predictive method is good practice, it is not easy for us to do so due to the limitations of our resources and the complexity of this new method. We will establish a web server for this method in the future. The most important part of this work is to introduce a U-shaped weight-transfer function into the pseudo-amino acid compositions. Since the signaling peptide information is useful in analyzing many different subcellular processes and this is the first time that the signaling peptide information has been emphasized in pseudo-amino acid composition representations, we believe that our method has great potential for application in predicting various attributes of proteins.

## Data Availability Statement

In this study, we took the ERPred dataset as our benchmarking dataset. Kumar et al released this dataset, along with their ERPred study (Kumar et al., 2017).

## Author Contributions

Y-YM curated the dataset, designed the algorithm, implemented the algorithm, and partially calibrated the parameters. WZ performed the experiments, partially calibrated the parameters, and collected the results. G-PL partially collected the results and analyzed the results. YG and P-FD investigated the question, designed the whole study, conceptualized the algorithm, analyzed the results, and wrote the manuscript.

## Funding

This work was supported by National Key R&D Program of China (2018YFC0910405); National Natural Science Foundation of China (NSFC 61872268); and Open Project Funding of CAS Key Lab of Network Data Science and Technology, Institute of Computing Tech-nology, Chinese Academy of Sciences (CASNDST201705).

## Conflict of Interest

The authors declare that the research was conducted in the absence of any commercial or financial relationships that could be construed as a potential conflict of interest.

## Supplementary Material

Table S1 Training Dataset.

Table S2 Independent testing Dataset-Positive.

Table S3 Independent testing Dataset-Negative.

## References

[B1] Almagro Armenteros J. J. Sønderby C. K. Sønderby S. K. Nielsen H. Winther O. (2017). DeepLoc: prediction of protein subcellular localization using deep learning. Bioinformatics 33, 3387–3395. 10.1093/bioinformatics/btx431 29036616

[B2] Araki K. Nagata K. (2011). Protein folding and quality control in the ER. Cold Spring Harb. Perspect. Biol. 3, a007526. 10.1101/cshperspect.a007526 21875985 PMC3220362

[B3] Briesemeister S. Rahnenführer J. Kohlbacher O. (2010). Going from where to why–interpretable prediction of protein subcellular localization. Bioinformatics 26, 1232–1238. 10.1093/bioinformatics/btq115 20299325 PMC2859129

[B4] Chen W. Feng P. Liu T. Jin D. (2019). Recent advances in machine learning methods for predicting heat shock proteins. Curr. Drug Metab. 20, 224–228. 10.2174/1389200219666181031105916 30378494

[B5] Chou K.-C. Shen H.-B. (2007). Euk-mPLoc: a fusion classifier for large-scale eukaryotic protein subcellular location prediction by incorporating multiple sites. J. Proteome Res. 6, 1728–1734. 10.1021/pr060635i 17397210

[B6] Chou K.-C. Shen H.-B. (2008). Cell-PLoc: a package of Web servers for predicting subcellular localization of proteins in various organisms. Nat. Protoc. 3, 153–162. 10.1038/nprot.2007.494 18274516

[B7] Chou K.-C. Shen H.-B. (2010). A new method for predicting the subcellular localization of eukaryotic proteins with both single and multiple sites: Euk-mPLoc 2.0. PloS One 5, e9931. 10.1371/journal.pone.0009931 20368981 PMC2848569

[B8] Chou K. C. Zhang C. T. (1995). Prediction of protein structural classes. Crit. Rev. Biochem. Mol. Biol. 30, 275–349. 10.3109/10409239509083488 7587280

[B9] Chou K.-C. Wu Z.-C. Xiao X. (2011). iLoc-Euk: a multi-label classifier for predicting the subcellular localization of singleplex and multiplex eukaryotic proteins. PloS One 6, e18258. 10.1371/journal.pone.0018258 21483473 PMC3068162

[B10] Chou K. C. (2001). Prediction of protein cellular attributes using pseudo-amino acid composition. Proteins 43, 246–255. 10.1002/prot1035 11288174

[B11] Chou K.-C. (2005). Using amphiphilic pseudo amino acid composition to predict enzyme subfamily classes. Bioinformatics 21, 10–19. 10.1093/bioinformatics/bth466 15308540

[B12] Chou K.-C. (2011). Some remarks on protein attribute prediction and pseudo amino acid composition. J. Theor. Biol. 273, 236–247. 10.1016/j.jtbi.2010.12.024 21168420 PMC7125570

[B13] Chou K.-C. (2013). Some remarks on predicting multi-label attributes in molecular biosystems. Mol. Biosyst. 9, 1092–1100. 10.1039/c3mb25555g 23536215

[B14] Chou K.-C. (2015). Impacts of bioinformatics to medicinal chemistry. Med. Chem. 11, 218–234. 10.2174/1573406411666141229162834 25548930

[B15] Dong Q. Zhou S. Guan J. (2009). A new taxonomy-based protein fold recognition approach based on autocross-covariance transformation. Bioinformatics 25, 2655–2662. 10.1093/bioinformatics/btp500 19706744

[B16] Du P. Xu C. (2013). Predicting multisite protein subcellular locations: progress and challenges. Expert Rev. Proteomics 10, 227–237. 10.1586/EPR.13.16 23777214

[B17] Du P. Yu Y. (2013). SubMito-PSPCP: predicting protein submitochondrial locations by hybridizing positional specific physicochemical properties with pseudoamino acid compositions. BioMed. Res. Int. 263829. 10.1155/2013/263829 PMC3763570 24027753

[B18] Du P. Li T. Wang X. (2011). Recent progress in predicting protein sub-subcellular locations. Expert Rev. Proteomics 8, 391–404. 10.1586/EPR.11.20 21679119

[B19] Ellgaard L. Helenius A. (2003). Quality control in the endoplasmic reticulum. Nat. Rev. Mol. Cell Biol. 4, 181–191. 10.1038/nrm1052 12612637

[B20] Huang W.-L. Tung C.-W. Huang H.-L. Hwang S.-F. Ho S.-Y. (2007). ProLoc: prediction of protein subnuclear localization using SVM with automatic selection from physicochemical composition features. BioSystems 90, 573–581. 10.1016/j.biosystems.2007.01.001 17291684

[B21] Huang W.-L. Tung C.-W. Ho S.-W. Hwang S.-F. Ho S.-Y. (2008). ProLoc-GO: utilizing informative Gene Ontology terms for sequence-based prediction of protein subcellular localization. BMC Bioinf. 9, 80. 10.1186/1471-2105-9-80 PMC2262056 18241343

[B22] Jiao Y. Du P. (2016). Performance measures in evaluating machine learning based bioinformatics predictors for classifications. Quant. Biol. 4, 320–330. 10.1007/s40484-016-0081-2

[B23] Jiao Y.-S. Du P.-F. (2017). Predicting protein submitochondrial locations by incorporating the positional-specific physicochemical properties into Chou’s general pseudo-amino acid compositions. J. Theor. Biol. 416, 81–87. 10.1016/j.jtbi.2016.12.026 28077336

[B24] Kabuss R. Ashikov A. Oelmann S. Gerardy-Schahn R. Bakker H. (2005). Endoplasmic reticulum retention of the large splice variant of the UDP-galactose transporter is caused by a dilysine motif. Glycobiology 15, 905–911. 10.1093/glycob/cwi085 15932921

[B25] Kumar R. Kumari B. Kumar M. (2017). Prediction of endoplasmic reticulum resident proteins using fragmented amino acid composition and support vector machine. Peer J. 5, e3561. 10.7717/peerj.3561 28890846 PMC5588793

[B26] Lavoie C. Paiement J. (2008). Topology of molecular machines of the endoplasmic reticulum: a compilation of proteomics and cytological data. Histochem. Cell Biol. 129, 117–128. 10.1007/s00418-007-0370-y 18172663 PMC2228376

[B27] Liao Z. Li D. Wang X. Zou* L. L. Q. (2018). Cancer diagnosis through IsomiR expression with machine learning method. Curr. Bioinf. 13 (1), 57–63. 10.2174/1574893611666160609081155

[B28] Lin H.-N. Chen C.-T. Sung T.-Y. Ho S.-Y. Hsu W.-L. (2009). Protein subcellular localization prediction of eukaryotes using a knowledge-based approach. BMC Bioinf. 10 Suppl 15, S8. 10.1186/1471-2105-10-S15-S8 PMC2788359 19958518

[B29] Lin W.-Z. Fang J.-A. Xiao X. Chou K.-C. (2013). iLoc-Animal: a multi-label learning classifier for predicting subcellular localization of animal proteins. Mol. Biosyst. 9, 634–644. 10.1039/C3MB25466F 23370050

[B30] Meng C. Jin S. Wang L. Guo F. Zou Q. (2019a). AOPs-SVM: a sequence-based classifier of antioxidant proteins using a support vector machine. Front. Bioeng. Biotechnol. 7, 224. 10.3389/fbioe.2019.00224 31620433 PMC6759716

[B31] Meng C. Wei L. Zou Q. (2019b). SecProMTB: support vector machine-based classifier for secretory proteins using imbalanced data sets applied to mycobacterium tuberculosis. Proteomics 19, 1900007. 10.1002/pmic.201900007 31348610

[B32] Nakai K. Horton P. (1999). PSORT: a program for detecting sorting signals in proteins and predicting their subcellular localization. Trends Biochem. Sci. 24, 34–36. 10.1016/S0968-0004(98)01336-X 10087920

[B33] Nakai K. Kanehisa M. (1991). Expert system for predicting protein localization sites in gram-negative bacteria. Proteins 11, 95–110. 10.1002/prot.340110203 1946347

[B34] Paschen W. Frandsen A. (2001). Endoplasmic reticulum dysfunction–a common denominator for cell injury in acute and degenerative diseases of the brain? J. Neurochem. 79, 719–725. 10.1046/j.1471-4159.2001.00623.x 11723164

[B35] Ryngajllo M. Childs L. Lohse M. Giorgi F. M. Lude A. Selbig J. (2011). SLocX: predicting subcellular localization of arabidopsis proteins leveraging gene expression data. Front. Plant Sci. 2, 43. 10.3389/fpls.2011.00043 22639594 PMC3355584

[B36] Shen H.-B. Chou K.-C. (2007). Hum-mPLoc: an ensemble classifier for large-scale human protein subcellular location prediction by incorporating samples with multiple sites. Biochem. Biophys. Res. Commun. 355, 1006–1011. 10.1016/j.bbrc.2007.02.071 17346678

[B37] Shen H.-B. Chou K.-C. (2009). A top-down approach to enhance the power of predicting human protein subcellular localization: Hum-mPLoc 2.0. Anal. Biochem. 394, 269–274. 10.1016/j.ab.2009.07.046 19651102

[B38] Stornaiuolo M. Lotti L. V. Borgese N. Torrisi M.-R. Mottola G. Martire G. (2003). KDEL and KKXX retrieval signals appended to the same reporter protein determine different trafficking between endoplasmic reticulum, intermediate compartment, and Golgi complex. Mol. Biol. Cell 14, 889–902. 10.1091/mbc.e02-08-0468 12631711 PMC151567

[B39] Teasdale R. D. Jackson M. R. (1996). Signal-mediated sorting of membrane proteins between the endoplasmic reticulum and the golgi apparatus. Annu. Rev. Cell Dev. Biol. 12, 27–54. 10.1146/annurev.cellbio.12.1.27 8970721

[B40] Verkhratsky A. (2002). The endoplasmic reticulum and neuronal calcium signalling. Cell Calcium 32, 393–404. 10.1016/S0143416002001896 12543098

[B41] Wan S. Mak M.-W. Kung S.-Y. (2012). mGOASVM: multi-label protein subcellular localization based on gene ontology and support vector machines. BMC Bioinf. 13, 290. 10.1186/1471-2105-13-290 PMC3582598 23130999

[B42] Wan S. Mak M.-W. Kung S.-Y. (2014). HybridGO-Loc: mining hybrid features on gene ontology for predicting subcellular localization of multi-location proteins. PloS One 9, e89545. 10.1371/journal.pone.0089545 24647341 PMC3960097

[B43] Wan S. Duan Y. Zou Q. (2017). HPSLPred: an ensemble multi-label classifier for human protein subcellular location prediction with imbalanced source. Proteomics 17, 1700262. 10.1002/pmic.201700262 28776938

[B44] Wang Z. Zou Q. Jiang Y. Ju Y. Zeng X. (2014). Review of protein subcellular localization prediction. Curr. Bioinf. 9, 331–342. 10.2174/1574893609666140212000304

[B45] Wei L. Ding Y. Su R. Tang J. Zou Q. (2018). Prediction of human protein subcellular localization using deep learning. J. Parallel Distrib. Comput. 117, 212–217. 10.1016/j.jpdc.2017.08.009

[B46] Yu C.-S. Chen Y.-C. Lu C.-H. Hwang J.-K. (2006). Prediction of protein subcellular localization. Proteins 64, 643–651. 10.1002/prot.21018 16752418

[B47] Zhao W. Li G.-P. Wang J. Zhou Y.-K. Gao Y. Du P.-F. (2019). Predicting protein sub-Golgi locations by combining functional domain enrichment scores with pseudo-amino acid compositions. J. Theor. Biol. 473, 38–43. 10.1016/j.jtbi.2019.04.025 31051179

[B48] Zhou H. Yang Y. Shen H.-B. (2017). Hum-mPLoc 3.0: prediction enhancement of human protein subcellular localization through modeling the hidden correlations of gene ontology and functional domain features. Bioinformatics 33, 843–853. 10.1093/bioinformatics/btw723 27993784

